# Heparin-binding protein as a novel biomarker for sepsis-related acute kidney injury

**DOI:** 10.7717/peerj.10122

**Published:** 2020-10-14

**Authors:** Sahra Pajenda, Andreja Figurek, Ludwig Wagner, Daniela Gerges, Alice Schmidt, Harald Herkner, Wolfgang Winnicki

**Affiliations:** 1 Department of Medicine III, Division of Nephrology and Dialysis, Medical University of Vienna, Vienna, Austria; 2 Department of Emergency Medicine, Medical University of Vienna, Vienna, Austria

**Keywords:** Acute kidney injury, Heparin-binding protein, Sepsis, Biomarker, Granzyme A

## Abstract

**Background:**

Sepsis-related acute kidney injury (AKI) is associated with high morbidity and mortality among patients. Underlying pathomechanisms include capillary leakage and fluid loss into the interstitial tissue and constant exposure to pathogens results in activation of inflammatory cascades, organ dysfunction and subsequently organ damage.

**Methods:**

To identify novel factors that trigger sepsis-related acute kidney injury, plasma levels of Granzyme A, as representative of a lymphocyte-derived protease, and heparin-binding protein as indicator for neutrophil-derived mediators, were investigated retrospectively in 60 sepsis patients.

**Results:**

While no association was found between plasma levels of lymphocyte-derived Granzyme A and the incidence of sepsis-related AKI, sepsis patients with AKI had significantly higher plasma levels of heparin-binding protein compared to those without AKI. This applies both to heparin-binding protein peak values (43.30 ±  23.34 vs. 30.25 ±  15.63 pg/mL; *p* = 0.005) as well as mean values (27.93 ±  14.39 vs. 22.02 ±  7.65 pg/mL; *p* = 0.021). Furthermore, a heparin-binding protein cut-off value of 23.89 pg/mL was established for AKI diagnosis.

**Conclusion:**

This study identifies the neutrophil-derived heparin-binding protein as a valuable new biomarker for AKI in sepsis. Beyond the diagnostic perspective, this offers prospect for further research on pathogenesis of AKI and novel therapeutic approaches.

## Introduction

At intensive care units physicians are often confronted with critically ill patients suffering from acute kidney injury (AKI) (Lameire, Van Biesen & Vanholder, 2005; Schrier et al., 2004). Such a severe state of organ dysfunction is known to lead to high morbidity and mortality even following recovery and discharge from hospital stay. This is especially serious in patients with severe sepsis and septic shock (Bagshaw et al., 2008; Schrier & Wang, 2004). An important focus in the clinical and experimental work-up is to identify patients at risk for developing AKI in order to take adequate measures before organ damage results in end stage renal disease. Laboratory parameters as serum creatinine or urine output are late markers for kidney injury and indicate damage when more than 50% of glomerular filtration rate is affected (Lameire, Van Biesen & Vanholder, 2005). To date, many biomarkers (NGAL, IL-18, KIM-1, IGFBP7 and TIMP-2, DDRGK1, neprilysin) for early detection of AKI have been described, but so far have barely reached their way into clinical practice (Gonzalez & Vincent, 2012; Han et al., 2002; Kashani et al., 2013; Kashani, Cheungpasitporn & Ronco, 2017; Mishra et al., 2004; Neziri et al., 2016; Pajenda et al., 2015; Pajenda, Mechtler & Wagner, 2017; Parikh et al., 2006).

Biomarkers often represent only indicators of renal cell damage (Beker et al., 2018). In contrast, molecules involved in inducing the damaging insult are of much greater importance to be identified (Holub et al., 2018; Oberbauer, 2008). Inactivation and elimination of such toxic compounds originating mostly from endogenous production would be the highest priority. Among those molecules inflicting damage at the renal microvascular structures and the proximal tubule are interleukins and proteases as well as neutrophil extracellular traps (NET) (Denning et al., 2019; Holub et al., 2018; Kumar et al., 2019). Many of them are secreted from effector cells and act as eliminators against the invading pathogens (Brinkmann et al., 2004). These effector cells include cytotoxic lymphocytes, which are known effectors against viral infections and tumor cells, and granulocytes, which act mainly against bacterial and fungal invaders (Fan & Zhang, 2005; Schepers, Arens & Schumacher, 2005; Teng et al., 2017).

Cytotoxic lymphocytes possess cytoplasmic granules containing cytolytic molecules. These are released to eliminate virus infected cells. A group of these granular proteins are proteases named granzymes (Wowk & Trapani, 2004). These enzymes are members of a cytolytic cascade of which the currently most accepted view is that granzymes can enter the target cells via a pore generated by perforin. This process is mediated through a conjugation of effector and target cell, thereby this interface effector molecules are delivered from the T-cell and NK cell onto the target cell (Blink, Trapani & Jans, 1999; Fan & Zhang, 2005; Osinska, Popko & Demkow, 2014). Although these molecules should be only directed towards the target cell and not against surrounding tissue, it has become clear that granzymes can be detected in various body compartments such as blood or synovial fluid (Lauw et al., 2000; Spaeny-Dekking et al., 1998) and play an important role within infections due to cytomegalovirus, Dengue virus, Epstein Barr virus, HIV or autoimmune diseases such as rheumatoid arthritis (Aggarwal, Sharma & Bhatnagar, 2014; Bade et al., 2005; Juno et al., 2017; Lauw et al., 2000). However, no data are yet available for soluble granzymes in the blood of sepsis patients and their association with AKI.

Furthermore, granulocytes are packaged with highly active proteases and enzymes involved in microbicidal activities (Benarafa & Simon, 2017; Pham, 2006). The Heparin- binding protein (HBP), also known as azurocidin, which is secreted from vesicles of activated neutrophils, is of particular importance in this context (Iversen et al., 1997; Pereira, 1995). It has been shown to be a relevant determinant for the severity of the critical state in the course of sepsis (Fisher & Linder, 2017; Linder, Soehnlein & Akesson, 2010). Moreover, in murine models high concentration of HBP has been shown to lead to kidney injury by interstitial enrichment of protein aggregates and interstitial hemorrhage suggesting increased vascular damage (Fisher et al., 2017). Accordingly, HBP has been ascribed a predictive value in septic AKI (Tverring et al., 2017). It is suggested that HBP plays an important role in the pathogenesis of AKI through increased endothelial permeability, enhanced inflammatory state and cell cycle arrest (Honore et al., 2019; Tyden et al., 2017).

High levels of circulating proteases can activate and damage capillary endothelial cells, especially at the glomeruli of the kidney and represent a crucial factor in the pathogenesis of AKI. Thus, in this study we focused on sepsis patients to determine levels of excreted Granzyme A (GrA) as well as HBP in plasma and AKI.

## Materials and Methods

### Study design

This cross-sectional retrospective study was carried out to determine plasma levels of Granzyme A (GrA) and heparin-binding protein (HBP) in sepsis patients. We further compared Granzyme A and heparin-binding protein levels in sepsis patients without AKI and sepsis patients with AKI stage I, II or III according to the KDIGO definition.

### Study population

Plasma samples of 60 patients above the age of 18 years fulfilling sepsis criteria with clinical records collected at the Medical University of Vienna were analyzed in this study.

Sepsis was defined by established sepsis criteria (Funk, Sebat & Kumar, 2009). Diagnosis of AKI was based on the KDIGO criteria using serum creatinine and urine output (Kellum, Lameire & Group, 2013). The initial sample collection was performed between 11/2010 and 11/2011 at the Department of Medicine III at the Medical University of Vienna.

The criteria for exclusion were an age under 18 years, present end-stage renal disease requiring renal replacement therapy and HIV or hepatitis C infections.

### Sample collection

Venous blood samples were collected from 60 patients; 43 sepsis patients with AKI and 17 gender and age-matched sepsis patients without AKI as controls. The first blood sample was taken within the first 24 h after study enrolment. Further samples were collected every 24 h, preferably in the morning, on in total 6 consecutive days wherever possible. Plasma probes were processed and stored in several aliquots at −80 °C within 30 min after blood collection. Repetitive freeze and thaw cycles of the plasma probes were avoided. Analytical staff was blinded by the use of bar codes.

### Plasma Granzyme A (GrA) measurement

The concentration of plasma granzyme A was determined using the Granzyme A Human ELISA (BioVendor, Brno, Czech Republic) following the manufacturer protocol. In brief: human EDTA plasma samples were thawed and centrifuged before the test procedure. 50 µL of patient plasma was mixed with 50 µL sample dilution fluid and applied onto the pre-coated multiwell dish. The provided recombinant protein was diluted in a standard series and similarly applied to the dish. After 2 h incubation at room temperature under constant shaking the plate was washed 3 times with the provided wash buffer, using an automated plate washer machine. Thereafter, the detection antibody was diluted in the provided fluid and 100 µL were applied onto the plate and incubated for 90 min at room temperature under constant shaking. After a second washing step the Streptavidin-HRP-conjugate was applied to the plate and incubated for 30 min. As a next step, following a final washing (five times), the provided developer solution was incubated for 20 min and then stopped with 50 µL stop solution. The colorimetric reading was performed at an ELISA reader at 405 nm and calculated according to the standard curve.

### Plasma heparin-binding protein (HBP)/Azurocidin measurement

For measurement of the heparin-binding protein/azurocidin the Human Azurocidin ELISA Kit (Abcam, Cambridge, UK) was performed according to the manual’s instruction. In brief, 100 µL of human EDTA plasma (diluted 1:11 in sample dilution buffer) was pipetted onto the ELISA plate and incubated for 90 min at 37 °C following shaking. Afterwards the plate was washed with TPBS washing buffer in an ELISA washing machine and 100 µL of the 1:100 diluted biotinylated anti-human azurocidin antibody was pipetted into each well. Following a 60 min incubation period at 37 °C the plate was washed 3 times in the ELISA washing machine using TPBS as wash buffer. One hundred µL of 1:100 diluted ABC (avidin-biotin-peroxidase complex) reagent in ABC dilution buffer was dispensed into each well and reacted at 37 °C for 30 min. After 5 washes with TPBS solution 90 µL of TMB substrate chromogen mixture was applied to the plate, reacted for 10 min at 37 °C and was stopped with 100 µL stop solution. The color reaction was read on an ELISA plate reader at 405 nm, whereas concentrations were calculated according to the standard series present on each individual ELISA plate.

### Laboratory diagnostics

All of the lab parameters have been analyzed at the Department of Laboratory Medicine of the Medical University of Vienna, where an accredited (ISO 15189:2008) and certified (ISO 9001:2008) quality management system is operated. The chronic kidney disease EPI (CKD-EPI) equation was used to determine baseline eGFR (Levey et al., 2009). Baseline eGFR values were based on serum creatinine values prior to sepsis and AKI, which were derived from the medical database.

### Outcomes

In this study the association between plasma levels of granzyme A and HBP in relation to AKI stage in sepsis patients was investigated.

### Data processing and statistical analyses

MS Excel (Microsoft, Redmont, WA, USA), Stata 14 for Mac (Stata Corp, College Station, TX, USA) and GraphPad Prism (GraphPad Prism version 7.00 for Windows, GraphPad Software, La Jolla California, USA) were used to manage and analyze the data. Continuous data are represented as mean ± standard deviation, categorized data are reported as absolute count and relative frequencies and sample size was estimated based on the planned regression analysis. To compare differences in the outcomes between the AKI categories on the original scale we applied the median test or the Fisher’s exact test, as appropriate. Granzyme A and Heparin-binding protein were log normally distributed. For regression analysis we used log-transformed Granzyme A and HBP as the dependent variables in the linear models. Co-variables were gender (male vs. female), age (years), peak serum C-reactive protein and peak white blood count. Estimates from the regression analysis are reported with 95% confidence interval and corresponding *P*-values. Standard metrics of diagnostic test accuracy with sensitivity and specificity of HBP levels (index test) were calculated to independently identify patients with AKI (reference standard). The optimal cut-off was estimated by maximizing the sensitivity/specificity product in accordance to Liu’s method (Liu, 2012) applying the adjustment method by Fluss (Fluss, Faraggi & Reiser, 2005). We estimated the confidence interval for the cut-off value by bootstrapping.

All tests performed were two-sided, *P*-values of less than 0.05 were considered significant.

### Data availability

Data sets that have been generated within this study are provided as Supplemental File.

### Ethics approval and consent to participate

Experimental protocols were all conducted in accordance to the Helsinki Declaration and approved by the ethics Committee of the Medical University of Vienna (EK 721/2007). Blood probes were collected from adult subjects above 18 years of age giving written informed consent. All methods were conducted according to relevant regulations and guidelines.

## Results

### Study population

The present study assessed demographics, laboratory data and plasma Granzyme A and Heparin-binding protein levels, detected by ELISA assays, of 43 sepsis patients with AKI (29 patients with AKI stage I, 6 patients with AKI stage II and 8 patients with AKI stage III) and 17 sepsis patients without AKI as controls. The demographic and clinical information of the whole study population is presented in Table 1.

**Table 1:** Baseline characteristics of the study population.

**Characteristic**	**Study subjects (*n* = 60)**
**Age (years)**	60.8 ± 16.6
**Gender - number (%)**	
- Male sex	39 (65)
- Female sex	21 (35)
**Unit of admission - number (%)**
- Intensive care unit	46 (76.7)
- Intermediate care unit	14 (23.3)
**Origin of infection - number (%)**	
- Respiratory tract	40 (66.7)
- Other	20 (33.3)
**Laboratory parameters:**	
- Peak serum C-reactive protein (mg/dL)	19.78 ± 9.82
- Peak white blood count (G/L)	16.18 ± 10.70
**Kidney function parameters:**	
- Baseline serum creatinine (mg/dL)	1.15 ± 0.72
- Baseline eGFR (mL/min)	76.20 ± 32.23
- Urine output (mL/24 h)	1023 ± 931
**Acute Kidney Injury (Kellum et al.) - number (%)**
- no AKI	17 (28.3)
- AKI stage I	29 (48.3)
- AKI stage II	6 (10.0)
- AKI stage III	8 (13.4)

**Notes.**

Plus-minus values are means ±  standard deviation. Numbers in parentheses indicate percentage.

Abbreviations AKI acute kidney injury eGFR estimated glomerular filtration rate KDIGO Kidney Disease: Improving Global Outcomes

### Plasma Granzyme A levels according to kidney function

Plasma samples from 60 patients were available for GrA testing at various consecutive time points during the course of sepsis. In total 237 GrA analyses were performed.

There was no association between GrA levels [pg/mL] in sepsis patients with AKI and without AKI. This was shown for peak GrA levels (22.77 ± 19.35 vs. 25.37 ± 33.21 pg/mL; *p* = 0.619) as well as mean GrA levels (16.14 ± 12.71 vs. 14.60 ± 17.38 pg/mL; *p* = 0.497). Furthermore, no difference was found between plasma GrA levels of patients with different stages of AKI (Table 2, Fig. S1).

**Table 2:** Clinical characteristics and plasma levels of AKI biomarkers of study patients.

	**Patients without AKI**	**Patients with AKI**	**AKI I**	**AKI II**	**AKI III**	***P*-value^a^**
Number of subjects	17	43	29	6	8	
**Characteristics**						
- Age (years)	60.8 ± 18.0	61.1 ± 16.0	62.5 ± 16.5	58.8 ± 13.8	57.8 ± 14.9	0.676
- Gender - male number (%)	10 (58.8)	29 (67.4)	20 (69.0)	4 (66.7)	5 (62.5)	0.560
**Laboratory parameters**						
- Peak serum C-reactive protein (mg/dL)	15.15 ± 8.84	21.08 ± 9.78	20.73 ± 9.41	23.74 ± 13.35	20.38 ± 7.3	0.144
- Peak white blood count (G/L)	18.30 ± 9.28	15.28 ± 11.26	14.09 ± 10.47	13.25 ± 6.75	21.14 ± 14.39	0.110
**Baseline kidney function parameters**						
- Serum creatinine (mg/dL)	0.96 ± 0.60	1.25 ± 0.75	1.10 ± 0.71	1.29 ± 0.59	1.77 ± 0.73	0.020
- Urine output (mL/24 h)	1,382 ± 966	895 ± 907	1,021 ± 957	841 ± 860	476 ± 562	0.045
**Clinical Course**						
- Survived - number (%)	16 (94)	30 (69.8)	23 (79.3)	5 (83.3)	2 (25.0)	0.050
- Need for RRT - number (%)	0 (0)	7 (16.3)	0 (0)	0 (0)	7 (87.5)	0.175
**Plasma GrA levels (pg/mL)**						
- peak GrA levels	25.37 ± 33.21	22.77 ± 19.35	22.81 ± 21.50	16.20 ± 11.02	22.61 ± 9.83	0.619
- mean GrA levels	14.60 ± 17.38	16.14 ± 12.71	15.91 ± 14.25	22.75 ± 17.85	16.89 ± 6.34	0.497
**Plasma HBP levels (pg/mL)**						
- peak HBP levels	30.25 ± 15.63	43.30 ± 23.34	36.38 ± 17.68	42.29 ± 21.76	64.81 ± 26.81	0.005
- mean HBP levels	22.02 ± 7.65	27.93 ± 14.39	24.46 ± 10.57	26.68 ± 10.61	39.22 ± 19.88	0.021

**Notes.**

Plus-minus values are means ±  standard deviation. Numbers in parentheses indicate percentage.

Abbreviations AKI acute kidney injury eGFR estimated glomerular filtration rate GrA Granzyme A HBP heparin-binding protein RRT renal replacement therapy

a *P*-value of patients without AKI vs. patients with AKI.

Multiple regression analysis revealed no association between GrA (peak as well as mean levels) and gender, age, peak serum C-reactive protein, peak white blood count (Table S1). Furthermore, there was no association between peak GrA as well as mean GrA levels and need for ICU admission (*p* = 0.579 or 0.364), treatment with vasopressors (*p* = 0.613 or 0.467), mechanical ventilation (*p* = 0.251 or 0.217) and resuscitation ( *p* = 0.484 or 0.675).

### Plasma Heparin-binding protein/Azurocidin levels according to kidney function

For the HBP testing plasma samples from 42 subjects were available at different successive time points in the course of their sepsis period. In total 156 HBP analyses were performed.

**Figure 1:**
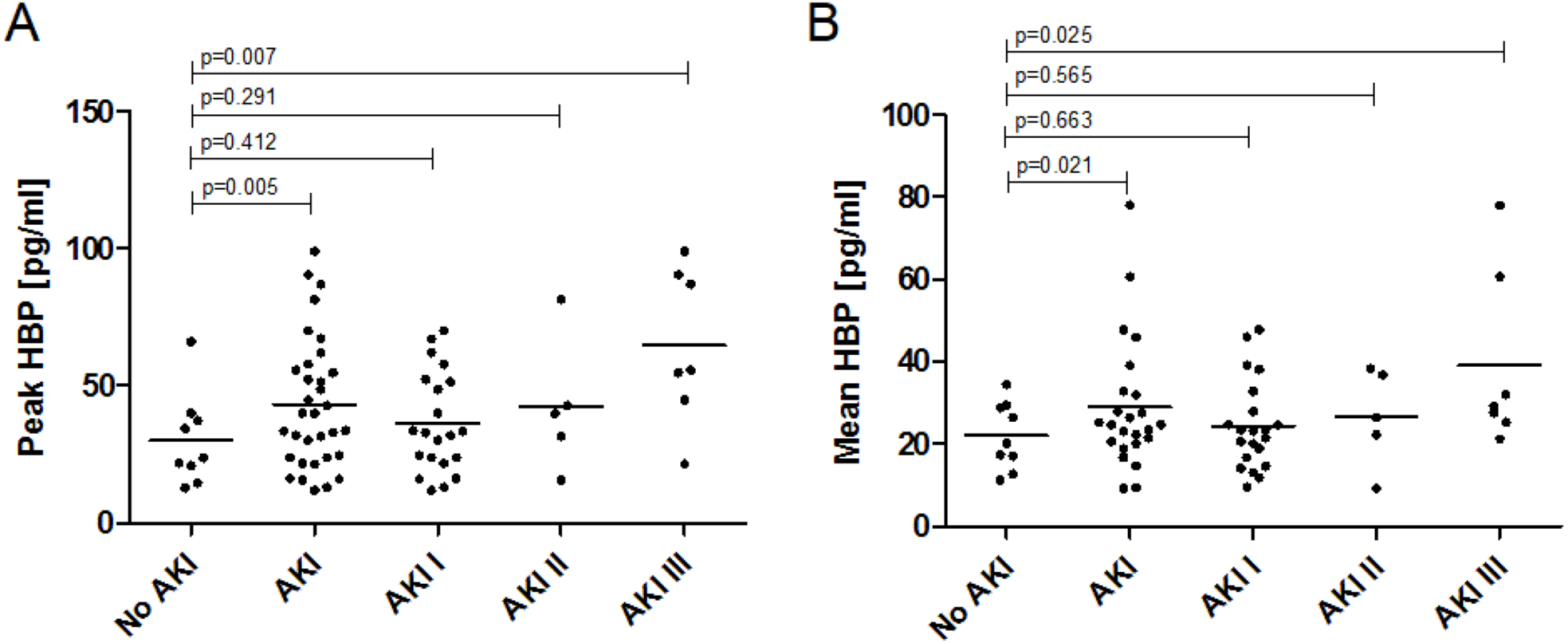
Plasma levels of Heparin-binding Protein in sepsis patients with and without acute kidney injury. (A) Peak plasma HBP levels and association with AKI stage. Peak HBP levels of patients with AKI in general (43.30 ± 23.34 pg/mL; *p* = 0.005) and AKI stage III (64.81 ± 26.81 pg/mL; *p* = 0.007) were significantly higher than in patients without AKI (30.25 ± 15.63 pg/mL). (B) Mean plasma HBP levels and association with AKI stage. Mean HBP levels of patients with AKI in general (22.02 ± 7.65 vs. 27.93 ± 14.39 pg/mL; *p* = 0.021) and AKI stage III (39.22 ± 19.88 pg/mL; *p* = 0.025) were significantly higher than in patients without AKI (22.02 ± 7.65 pg/mL).

In general, sepsis patients with AKI presented higher plasma HBP levels compared to sepsis patients without AKI. This was shown for peak HBP levels (43.30 ± 23.34 vs. 30.25 ± 15.63 pg/mL; *p* = 0.005) as well as mean HBP levels (27.93 ± 14.39 vs. 22.02 ± 7.65 pg/mL; *p* = 0.021). Plasma levels of HBP were significantly highest in AKI stage III, this is true for both peak and mean HBP levels (Table 2, Fig. 1). The HBP levels were log normally distributed. With each increase in AKI stage the coefficient for the increase in log peak HBP was 0.25 (95% CI [0.08–0.421]; *p* = 0.005) and 0.17 (95% CI [0.03–0.313]; *p* = 0.021) for the increase in log mean HBP.

In the course of sepsis and AKI HBP levels showed a tendency towards higher values within the first days of disease with some variability over time (day 1: 31.4 ± 19.8 pg/mL, day 2: 26.2 ± 20.4 pg/mL, day 3: 34.5 ± 20.5 pg/mL, day 4: 26.0 ± 18.6 pg/mL, day 5: 24.6 ± 17.5 pg/mL, day 6: 27.6 ± 22.7 pg/mL) (Fig. S2).

Also for HBP the multiple regression analysis revealed no association between HBP (peak as well as mean levels) and gender, age, peak serum C-reactive protein, peak white blood count. (Table 3). Furthermore, there was no association between peak HBP as well as mean HBP levels and need for ICU admission (*p* = 0.595 or 0.804), treatment with vasopressors (*p* = 0.605 or 0.861), mechanical ventilation (*p* = 0.486 or 0.758) and resuscitation (*p* = 0.727 or 0.970).

### Sensitivity and specificity of plasma Heparin-binding protein for AKI diagnosis

A receiver operating characteristic (ROC) analysis was conducted to assess whether plasma HBP is a valid indicator for diagnosis and differentiation of AKI.

**Figure 2:**
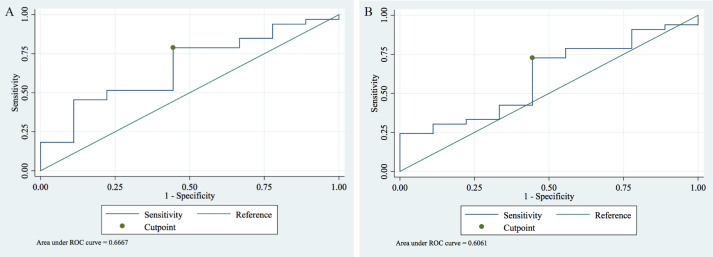
Receiver operating characteristic (ROC) analysis of the plasma HBP in sepsis patients with and without AKI. (A) For peak HBP the cut-off value of 23.89 pg/mL had a sensitivity of 0.79 and a specificity of 0.56 with an AUC of 0.67 (95% CI [0.46–0.87]) to detect AKI. (B) For mean HBP the cut-off value of 20.46 pg/mL had a sensitivity and specificity of 0.73 and 0.56, respectively, with an AUC of 0.61 (95% CI [0.40–0.81]) to detect AKI.

We identified that the optimal peak HBP cut-off value is 23.89 pg/mL (95% CI [7.07–40.72]) for discrimination between AKI vs. non-AKI with a sensitivity and specificity of the test of 0.79 and 0.56 and an AUC of 0.67 (95% CI [0.46–0.87]) (Fig. 2A).

**Table 3:** Association of clinical variables and parameters with plasma HBP levels in sepsis patients.

**Variable**	**Regression coefficient** **(95% CI) with peak HBP** **adjusted**^a^	*P*-value	**Regression coefficient** **(95% CI) with mean HBP** **adjusted**^a^	*P*-value
Gender (male)	−0.155 (−0.514 to 0.203)	0.385	−0.071 (−0.036 to 0.222)	0.625
Age (years)	−0.001 (−0.012 to 0.010)	0.880	−0.005 (−0.014 to 0.004)	0.261
Peak serum C-reactive protein (mg/dL)	−0.006 (−0.024 to 0.011)	0.467	−0.009 (−0.024 to 0.005)	0.177
Peak white blood count (G/L)	0.001 (−0-001 to 0.001)	0.899	0.001 (−0.001 to 0.001)	0.810

**Notes.**

a multivariable adjustment for gender (male/female), age (years), peak serum C-reactive protein and peak white blood count.

Abbreviations HBP heparin-binding protein

For mean HBP the optimal cut-off is 20.46 pg/mL (95% CI [8.53–32.39]) for AKI vs. non-AKI differentiation. For this cut-off value, the sensitivity and specificity of the test were 0.73 and 0.56 with an AUC of 0.61 (95% CI [0.40–0.81]) (Fig. 2B).

Our findings indicate that both peak HBP and mean HBP can be used to differentiate AKI from non-AKI with peak HBP showing higher levels of differentiation.

## Discussion

In this study we evaluate whether lymphocyte-derived GrA or neutrophil-derived HBP are indicators for acute kidney injury in the course of sepsis. Despite presence of natural inhibitors of proteases in serum, elevated levels of proteases in blood might be involved in activating endothelial cells in the microvasculature, leading to activation of an inflammatory cascade. In our study GrA levels showed no association with the incidence of AKI in sepsis patients. In contrast, HBP is significantly elevated in sepsis patients with AKI and is further associated with the severity of acute renal failure, with HBP levels highest in AKI stage III.

Hereby, it is to mention that in our study 7 out of 8 patients with AKI stage III required renal replacement therapy (RRT). In a previous study it was shown that although renal impairment may lead to higher renal clearance of HBP compared to healthy individuals, HBP levels were not significantly reduced by continuous RRT (Samuelsson et al., 2019). This observation is supported by our findings, where patients with AKI stage III undergoing RRT had the highest HBP levels.

Heparin-binding protein, as important indicator for neutrophil-derived substances in the blood circulation, has previously been studied in animal models with respect to AKI and was shown to be relevant in pathologies affecting the nephron in sepsis (Fisher et al., 2017). Although in some experiments no protease activity has been ascribed to this molecule, it represents an ideal indicator for neutrophil activation and degranulation (Tapper et al., 2002). This implies that also other proteases are released into the blood circulation when elevated HBP levels are detected. Much research has already been conducted on the pathomechanism of neutrophil-derived cell products and the initiation of cell-destructive processes (Bentzer et al., 2016; Gautam et al., 2001). On the one hand neutrophil extracellular traps (NET) have been shown to trigger a cascade via activation of coagulation processes and endothelial damage, leading to thrombotic microangiopathy (Arai et al., 2013; Fuchs et al., 2012). On the other hand, various protease sensitive receptors are expressed on the endothelial cell surface, which can lead to endothelial cell activation when cleaved (Gerszten et al., 1994). Endothelial cell activation entails an increase of permeability and fluid extravasation from the vascular bed. This is of particular importance for the microenvironment of the nephron, especially at the proximal tubule, where a disturbance of the fine-tuned equilibrium leads to impairment of fluid, electrolyte and substrate reabsorption. Disequilibrium at the proximal tubular cells may result in pathologies like loss of cell polarity, loss of brush border and cast formation associated with AKI (Lerolle et al., 2010).

For clinical practice this indicates that early intervention, before sepsis leads to excessive degranulation of neutrophils, may be of great importance to prevent cell damage.

The administration of heparin has been investigated in this context, however, its effectiveness is discussed, though it is plausible that negatively charged heparins can bind the strongly positively charged proteases (Fisher et al., 2017). It is however only partially conceivable that this strategy has beneficial effects, since proteases derived from neutrophils and natural killer cells are generally bound to negatively charged proteoglycans, which act as storage inactivators within the granular environment when still in inactive state within the effector cell (Masson et al., 1990). Moreover, apheresis of extracellular proteases via heparin columns or the administration of positively charged aprotinin could have positive effect on the protease inactivation and blood clearance of these potentially harmful biologically highly active neutrophil-derived molecules.

The major limitation of our study is the retrospective nature, which limited the number of analyses due to pre-defined study conditions and thus the availability of selectively obtained probes. In addition, no urine probes were obtained that might have enabled further valuable analyses. This is due to the fact that a number of patients presented with anuria and also because the assay used for HBP analyses is technically not approved for urine samples. Finally, GrA and HBP are activated and triggered differently by bacterial, viral and fungal pathogens. However, the heterogeneity and sample size available for this study did not allow stratification by the microbial source of sepsis.

Our study, though, was strengthened by a well characterized cohort of sepsis patients with and without AKI according to KDIGO definition, which resulted in clear results.

While GrA as representative of a lymphocyte-derived protease was not associated with the incidence of AKI in sepsis patients, significantly elevated levels of HBP, as an indicator protein of neutrophil origin, were detected in sepsis patients with AKI.

Previous studies have shown the molecular pathomechanisms of HBP involved in vascular leakage, chemotaxis and inflammation in the course of sepsis, cardiac and respiratory failure and various infections. It has been identified not only as a promising diagnostic marker, but also has prognostic relevance for severity of disease (Yang et al., 2019). However, the purpose of this study was not to determine the role of GrA and HBP in the pathogenesis of AKI during sepsis, but to investigate their significance in AKI diagnosis. Plasma levels of HBP tended to be higher within the first days of disease and showed a decreasing trend until day 6, which marked the end of our observation. Overall, HBP proved to be a robust diagnostic marker for AKI in our study. Hereby, we provide HBP cut-offs to detect AKI and demonstrate that HBP is a novel and useful AKI biomarker.

## Conclusion

Heparin-binding protein, as important indicator for neutrophil-derived mediators in the bloodstream, is a promising new biomarker for AKI in sepsis. It is not only of interest for diagnostics, but opens doors for future studies on pathogenesis and therapeutic approaches of AKI.

##  Supplemental Information

10.7717/peerj.10122/supp-1 Supplemental Information 1 Plasma levels of Granzyme A in sepsis patients with and without acute kidney injury (A) Peak plasma GrA levels and association with AKI stage. Peak GrA levels were not different between patients without AKI (25.37 ± 33.21 pg/mL) and with AKI (22.77 ± 19.35 pg/mL; *p* = 0.619) at any stage. (B) Mean plasma GrA levels and association with AKI stage. Also mean GrA levels were not different between patients without AKI (14.60 ± 17.38 pg/mL) and with AKI 16.14 ± 12.71 pg/mL; *p* = 0.497) at any stage.

10.7717/peerj.10122/supp-2 Supplemental Information 2 Course of Heparin-binding protein over time Plasma heparin-binding protein levels tended to be higher within the first days of disease and showed a decreasing trend with some variability over time: (day 1: 31.4 ± 19.8 pg/mL, day 2: 26.2 ± 20.4 pg/mL, day 3: 34.5 ± 20.5 pg/mL, day 4: 26.0 ± 18.6 pg/mL, day 5: 24.6 ± 17.5 pg/mL, day 6: 27.6 ± 22.7 pg/mL). Variability expressed as standard deviation ranged from 17.5 to 22.7 pg/mL on each day, the average intra-patient standard deviation was 13.1 pg/mL.

10.7717/peerj.10122/supp-3 Supplemental Information 3 Association of clinical variables and parameters with plasma Granzyme A levels in sepsis patients Abbreviations: GrA, Granzyme A. a multivariable adjustment for gender (male/female), age (years), peak serum C-reactive protein and peak white blood count.

10.7717/peerj.10122/supp-4 Supplemental Information 4 Raw Data

10.7717/peerj.10122/supp-5 Supplemental Information 5 Codebook for raw data

## Additional Information and Declarations

### Data Availability

The following information was supplied regarding data availability: The raw data and codebook are available in the Supplemental Files.
