# Supplementary figures and images for: Heparin-binding protein as a novel biomarker for sepsis-related acute kidney injury
Source: PeerJ. 2020 Oct 14;8:e10122. doi: 10.7717/peerj.10122 (PMC7568480; doi:10.7717/peerj.10122)

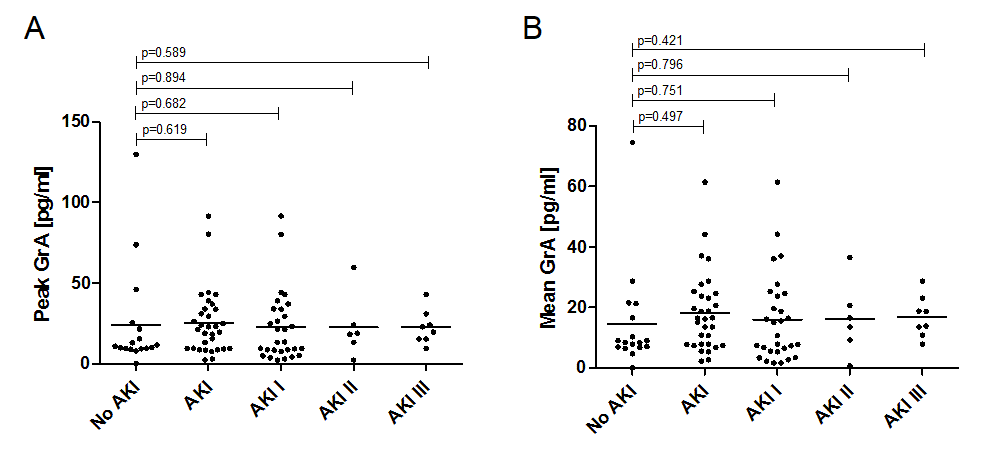

Supplement: Supplemental Information 1 — (A) Peak plasma GrA levels and association with AKI stage. Peak GrA levels were not different between patients without AKI (25.37 ± 33.21 pg/mL) and with AKI (22.77 ± 19.35 pg/mL; p = 0.619) at any stage. (B) Mean plasma GrA levels and association with AKI stage. Also mean GrA levels were not different between patients without AKI (14.60 ± 17.38 pg/mL) and with AKI 16.14 ± 12.71 pg/mL; p = 0.497) at any stage. [file peerj-08-10122-s001.png]

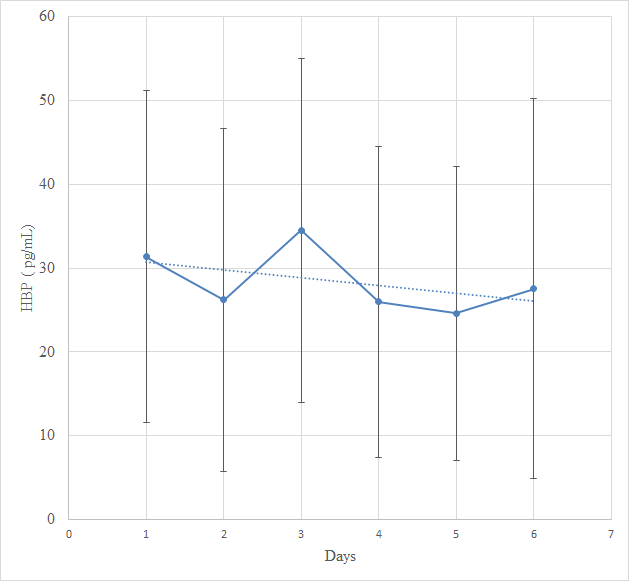

Supplement: Supplemental Information 2 — Plasma heparin-binding protein levels tended to be higher within the first days of disease and showed a decreasing trend with some variability over time: (day 1: 31.4 ± 19.8 pg/mL, day 2: 26.2 ± 20.4 pg/mL, day 3: 34.5 ± 20.5 pg/mL, day 4: 26.0 ± 18.6 pg/mL, day 5: 24.6 ± 17.5 pg/mL, day 6: 27.6 ± 22.7 pg/mL). Variability expressed as standard deviation ranged from 17.5 to 22.7 pg/mL on each day, the average intra-patient standard deviation was 13.1 pg/mL. [file peerj-08-10122-s002.png]
