# Supplementary material for: Heparin-binding protein as a novel biomarker for sepsis-related acute kidney injury
Source: PeerJ. 2020 Oct 14;8:e10122. doi: 10.7717/peerj.10122 (PMC7568480; doi:10.7717/peerj.10122)
Supplement: Supplemental Information 3 — Abbreviations: GrA, Granzyme A. a multivariable adjustment for gender (male/female), age (years), peak serum C-reactive protein and peak white blood count. [file peerj-08-10122-s003.docx]

**Supplemental Table 1. Association of clinical variables and parameters with plasma Granzyme A levels in sepsis patients.**

| **Variable** | **Regression coefficient**  **(95% CI) with peak GrA**  **adjusted**^a^ | **P-value** | **Regression coefficient**  **(95% CI) with mean GrA**  **adjusted**^a^ | **P-value** |
| --- | --- | --- | --- | --- |
| Gender (male) | -0.103 (-0.596 to 0.389) | 0.675 | -0.088 (-0.588 to 0.412) | 0.725 |
| Age (years) | -0.004 (-0.019 to 0.011) | 0.575 | -0.008 (-0.023 to 0.008) | 0.299 |
| Peak serum C-reactive protein (mg/dL) | -0.022 (-0.047 to 0.002) | 0.075 | -0.023 (-0.048 to 0.002) | 0.065 |
| Peak white blood count (G/L) | 0.001 (-0-001 to 0.001) | 0.995 | 0.001 (-0.001 to 0.001) | 0.768 |

Abbreviations: GrA, Granzyme A.

^a^ multivariable adjustment for gender (male/female), age (years), peak serum C-reactive protein and peak white blood count.
