# Supplementary material for: Heparin-binding protein as a novel biomarker for sepsis-related acute kidney injury
Source: PeerJ. 2020 Oct 14;8:e10122. doi: 10.7717/peerj.10122 (PMC7568480; doi:10.7717/peerj.10122)
Supplement: Supplemental Information 5 [file peerj-08-10122-s005.docx]

CODEBOOK for raw data

Gender

0 – female

1 – male

unit_ICU1_IMC0

0 – IMC

1 – ICU

infection_origin_1lung_2abd_3skin_4uti_5viral_6unkn

1 – lung infection

2 – abdominal infection

3 – skin infection

4 – urinary tract infection

5 – viral infection

6 – unknown

survivded

0 – did not survive

1 – survived

RRT

0 – no need for renal replacement therapy

1 – need for renal replacement therapy

cpr

0 – no cardiopulmonary resuscitation

1 – cardiopulmonary resuscitation

mechanical ventilation

0 – no need for mechanical ventilation

1 – need for mechanical ventilation

vasopressors

0 – no need for vasopressors

1 – need for vasopressors

aki stage

0 – no acute kidney injury

1 – acute kidney injury stage 1

2 – acute kidney injury stage 2

3 – acute kidney injury stage 3
